# BCI-Based Control for Ankle Exoskeleton T-FLEX: Comparison of Visual and Haptic Stimuli with Stroke Survivors

**DOI:** 10.3390/s21196431

**Published:** 2021-09-26

**Authors:** Patricio Barria, Angie Pino, Nicolás Tovar, Daniel Gomez-Vargas, Karim Baleta, Camilo A. R. Díaz, Marcela Múnera, Carlos A. Cifuentes

**Affiliations:** 1 Department of Electrical Engineering, University of Magallanes, Punta Arenas 6210427, Chile; pbarria@rehabilitamos.org; 2 Club de Leones Cruz del Sur Rehabilitation Center, Punta Arenas 6210133, Chile; kbaleta@rehabilitamos.org; 3 Brain-Machine Interface Systems Lab, Systems Engineering and Automation Department, Miguel Hernández University of Elche UMH, 03202 Elche, Spain; 4 Department of Biomedical Engineering, Colombian School of Engineering Julio Garavito, Bogotá 111166, Colombia; angie.pino-l@mail.escuelaing.edu.co (A.P.); bryan.tovar@mail.escuelaing.edu.co (N.T.); daniel.gomez-v@mail.escuelaing.edu.co (D.G.-V.); marcela.munera@escuelaing.edu.co (M.M.); 5 Institute of Automatics, National University of San Juan, San Juan 5400, Argentina; 6 Graduate Program in Electrical Engineering, Federal University of Espirito Santo, Vitoria 29075-910, Brazil; camilo.diaz@ufes.br

**Keywords:** brain–computer interface (BCI), beta rebound, central nervous system (CNS), electroencephalography (EEG), ankle exoskeleton, motor imagery (MI), visual stimulus, haptic stimulus

## Abstract

Brain–computer interface (BCI) remains an emerging tool that seeks to improve the patient interaction with the therapeutic mechanisms and to generate neuroplasticity progressively through neuromotor abilities. Motor imagery (MI) analysis is the most used paradigm based on the motor cortex’s electrical activity to detect movement intention. It has been shown that motor imagery mental practice with movement-associated stimuli may offer an effective strategy to facilitate motor recovery in brain injury patients. In this sense, this study aims to present the BCI associated with visual and haptic stimuli to facilitate MI generation and control the T-FLEX ankle exoskeleton. To achieve this, five post-stroke patients (55–63 years) were subjected to three different strategies using T-FLEX: stationary therapy (ST) without motor imagination, motor imagination with visual stimulation (MIV), and motor imagination with visual-haptic inducement (MIVH). The quantitative characterization of both BCI stimuli strategies was made through the motor imagery accuracy rate, the electroencephalographic (EEG) analysis during the MI active periods, the statistical analysis, and a subjective patient’s perception. The preliminary results demonstrated the viability of the BCI-controlled ankle exoskeleton system with the beta rebound, in terms of patient’s performance during MI active periods and satisfaction outcomes. Accuracy differences employing haptic stimulus were detected with an average of 68% compared with the 50.7% over only visual stimulus. However, the power spectral density (PSD) did not present changes in prominent activation of the MI band but presented significant variations in terms of laterality. In this way, visual and haptic stimuli improved the subject’s MI accuracy but did not generate differential brain activity over the affected hemisphere. Hence, long-term sessions with a more extensive sample and a more robust algorithm should be carried out to evaluate the impact of the proposed system on neuronal and motor evolution after stroke.

## 1. Introduction

Stroke is one of the leading causes of physical disability seriously affecting 5 million people’s quality of life out of the 15 million who suffer from stroke around the world [1]. About 80% of stroke survivors have residual mobility limitations usually associated with a foot-drop. That means a lower limb impairment that combines a weak dorsiflexor and an increased plantar flexor stiffness reducing the capacity to maintain balance and posture while walking [2,3,4]. Post-stroke rehabilitation therapy aims to restore the patient’s physical, neurological, and psychological capacities to achieve the highest level of functional independence [5]. In fact, robotic devices like lower-limb exoskeletons in motor rehabilitation programs have been shown to improve automatic repetitive training and promote new motor skill acquisition after stroke [6,7]. User’s intention in this field is usually detected and predicted through control approaches based on the sensing of human biomechanics (i.e., through inertial sensors, direct contact operation, or external transducers) [7,8]. Therefore, conventional robotic control systems generally do not include efficient and natural interaction methods between users and exoskeletons [9]. In this way, the possibility of enhancing and involve the patient increasingly is a clear objective to improve the user skills in a short-term period with better results.

Brain–computer interfaces (BCI), mostly based on the acquisition of electroencephalography (EEG) biological signals, provides a promising communication and control channel to improve the patient’s involvement with the system. It has been shown to generate neuroplasticity progressively throughout the development of neuromotor abilities and the mental practice of movements [10,11]. Besides, this technology has emerged as a potential tool to command robotic exoskeletons (e.g., lower-body powered exoskeletons) in the assistance and rehabilitation fields [9]. One of the few clinical studies exploring the BCI-based rehabilitation systems showed the viability of this tool based on motor-related events when a user is commanding a lower-limb exoskeleton [12]. Other research, also focused exclusively on the ankle-foot orthosis, showed a fast and effective approach for inducing cortical plasticity through BCI having a huge prospective in motor function rehabilitation after stroke [13].

In the case of the control of robotic exoskeletons by means of a BCI, several paradigms exists based on rhythms related to the brain activity [12]. One of the most used strategies to decode brain activity is the motor imagery (MI) study [14]. Motor imagery is a technique that requires a dynamic mental image of the desired motor output [15]. Its use in the BCI field has been relevant to detect neurological patients’ movement intention. Specifically throughout the Event-Related Desynchronization/Synchronization (ERD/ERS) modality, it is possible to recognize the beta band’s variations in power after performing a real or an imagined movement [16,17,18]. Generally, the alpha and beta power decrease in the resting state and keeps a reduced power during the motor imagination or planning (ERD). However, about 300 or 500 ms after the end of the motor imagery, the beta rebound emerges through one second approximately (ERS). This last event occurs particularly in motor areas representing a simple idle activity and/or an active inhibition of the motor network [19,20]. ERD/ERS pattern has been widely studied in MI-BCI modalities as a potentially effective strategy for detecting and measuring commands to control a system [21,22]. For instance, the beta cortical oscillations control signals were effective to actuate an upper-limb exoskeleton with motor execution and motor imagination [23], and to trigger a robot-assisted action during lower limb motor imagery tasks [24,25]. In particular, one of the inspirational developments in the BCI-based beta rebound system was used to control a virtual spaceship takeoff using real or imaginary foot movements. The design of this strategy resulted in effective commands that easily interpret neural signals as motor intentions to activate the animation in the virtual reality (VR) environment without MI training [26,27,28].

In general, motor imagery-based BCIs are commonly related to low performance and reliability due to imperfect signal processing algorithms and most users’ complexity to display a vivid picture of the movement [8,29]. According to Lotte et al. [30], the user, beyond the processing techniques, is one of the most critical components of the BCI loop. The subject’s inability to correctly perform the desired mental commands hinders the capacity of any algorithm to properly detect them [30]. In this sense, the user must be properly guided to be able to effectively use and control the BCI system [30,31,32]. Several sources agree the proper induction of MI is a suitable and beneficial alternative for patients in their rehabilitation process [33,34]. Precisely, MI-BCI strategies induce neural activity and increase motor and cognitive performance by generating a change in brain cortical activity [33,35]. In addition, the specific modulation of the brain for planning and control voluntary exoskeleton movements triggers neuroplasticity in post-stroke patients [36,37]. These neurofeedback mechanisms generate brain reorganization to restore the lost function and consequently prompt a motor recovery [36,38]. Some studies reinforce this idea by including other signals, cues, feedback systems, and even other modalities within the therapy protocol [39].

Motor imagery-associated stimuli has been considered as an effective strategy to proficiently regulating motor imagery [15]. Motivation and compatibility with the therapy have been increasingly involved in the BCI systems protocols making users possible to learn to regulate electrocortical activity in the sensorimotor cortex. Usually, visual stimulus is most applied in this field to support users’ motor imagery task [30]. Neuper et al. [40] have shown the control of an MI-based BCI system can develop a better precision in its performance through visual developments. Nevertheless, haptic stimulus has been reported to be more engaging and functional than the visual in MI-BCI systems [41,42]. According to Kauhanen et al. [43], haptic stimulus has emerged as complement to regulate motor imagery generation. In this way, MI-based BCI with a haptic stimulus can be an effective alternative when the visual channel is overloaded or when it is needed for the performance of additional tasks beyond the BCI system [41,43].

Following this line of research, this preliminary study seeks to develop a BCI-controlled ankle exoskeleton system based on motor imagery to activate neuronal and motor patterns in post-stroke recovery. Furthermore, this report looks to evaluate the best strategy to induce MI through a comparison of visual and visual with haptic stimuli modalities. From this, it is expected to introduce a complete and portable system to actively involve stroke survivors in robotic therapies. To do that, BCI motor imagination accuracy, offline EEG signal analysis, and user level of satisfaction are presented.

## 2. Materials and Methods

### 2.1. BCI-Exoskeleton System

This section presents the proposed main elements and procedures developed to control the T-FLEX ankle exoskeleton actuation by the BCI system implementation with visual and haptic stimulations. In this sense, the system integrated BCI wearable system with an EEG signal treatment for MI detection, followed by the T-FLEX robotic device assistance and the integrated protocols to communicate both the systems. Additionally, the system contained stimuli strategies with their respective operation technique and user presentation modes.

#### 2.1.1. BCI Interface

The interface included the flexible and wireless EEG Headset Enobio 20 (Neuroelectrics, Barcelona, Spain) which contained 20 channels, a 500 Hz frequency rate, and high dynamic resolution (24 bits, 0.05 uV). The Enobio Hardware linked with the NIC 2.0 Software (Neuroelectrics, Spain) operated the EEG signal acquisition system to allow the motor cortex recording [44]. Particularly, this study worked through a laplacian montage with 4 solidgel electrodes positioned according to the international 10/20 system distribution (C1, C2, FCz and CPz with the Cz reference electrode).

Meanwhile, OpenVibe Software (Inria Rennes, France) processed the real-time EEG signal through a pre-processing and a feature extraction stage, based on [16,26]. The pre-processing phase consisted of a Laplacian Spatial Filter and a 4th-order Butterworth band-pass filter (pass band ripple of 0.5 dB) with a lower and upper cutoff frequency of 16 and 24 Hz (the beta band), respectively [45,46]. Denoising and cleaning the signal eliminates artifacts product of the environment and the user’s physical conditions (e.g., skin impedance fluctuations, compensatory movements, muscle activity, eye movements, etc). Besides, combining filtering strategies create, as far as possible, an ideal signal with less noise where the data utility is maximized. In this case, the methodological development of the study focuses on performing a continuous measurement of the power of the beta rhythm in a Laplacian montage around Cz to detect lower-limb MI. The above is to assess the reliability of the robotic strategy combined with an MI-based control without beta laterality considerations [16,47]. Although the ERS beta rhythm is generally dominant over the contralateral primary sensorimotor area [20], discrepancies still exist related to its lateralization. According to Nam et al. [48], the MI of a limb movement can be accompanied by an ipsilateral or a contralateral ERS under the movement duration. Thus, brief movement imagery generates ipsilateral ERS while continuous movement imagery yields contralateral ERS [48].

That said, the beta power rebound identification considered an initial signal epoching into 1 s long epochs and 100 ms overlapping. Then, the signal passed through a square operation and average calculation over a 1 s interval. A 5-min calibration process defined a threshold (Th), computed as the average plus three times the standard deviation as indicated in Equation (1), where $\bar{x}$ is the average of the signal and σ is the standard deviation of the signal over the specified interval. Lastly, the online scenario compares the computed Th value to the real-time beta power signal to establish the beta rebound detection [16].

(1) $$Th=\bar{x}+3\sqrt{\sigma^{2}}\;$$

#### 2.1.2. T-FLEX Ankle Exoskeleton

**Figure 1:**
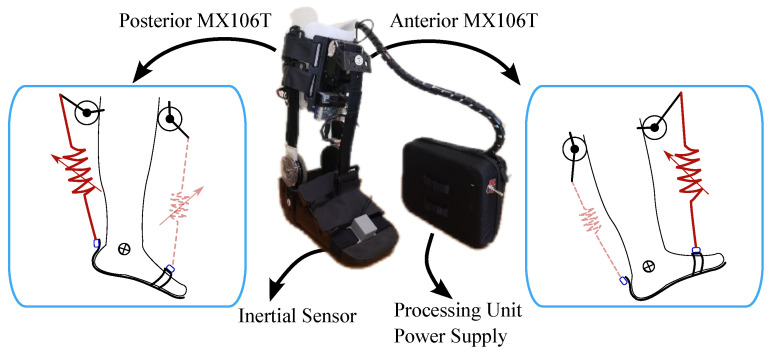
The actuation system of the T-FLEX exoskeleton implemented on a passive orthotic device. The left and right parts show the movements assisted by the device and the involved elements and actuators.

T-FLEX is a wearable and portable exoskeleton capable of assisting the ankle during stationary and gait assistance scenarios through a variable stiffness principle [49]. The device comprises two servomotors Dynamixel MX106T (Dynamixel, Seoul, Korea), placed on the user’s shank that emulate the human muscles (see Figure 1). These actuators employ elastic elements to transmit the torque to the ankle, whose mechanical behavior under stress tests is similar to the human Achilles tendon [50]. The T-FLEX’s operating principle consists of an agonist-antagonist configuration to assist the ankle motions in the sagittal plane. In this sense, the anterior actuator contributes to the dorsiflexion, and the posterior actuator provides the plantarflexion, as Figure 1 shows.

The exoskeleton integrates a low-cost small single-board computer (SBC-Raspberry Pi 3, Raspberry, UK) as the processing unit to control the actuators and acquire the device’s sensors, i.e., an inertial sensor BNO055 (Bosch, Stuttgart, Germany) to estimate the user’s ankle kinematics and motor data to measure user-device interaction. The controllers and algorithms run on the Robot Operating System framework under a Unix-based distribution and are available in a public repository at https://github.com/GummiExo/t_flex (accessed on 4 May 2020). The device includes a LiPo battery of 14.8 V and 4800 mAh that enables each actuator to turn to 55 rpm (no-load condition) and provide a stall torque of 10 Nm.

T-FLEX has shown promising results in (1) gait assistance and (2) stationary scenarios in terms of real applications involving stroke survivors [51,52]. Specifically, a study evidenced significant changes in motor recovery (i.e., improvement in dorsiflexion during the swing phase, spasticity reduction, and increase in walking speed and cadence) after 18 sessions of T-FLEX in a stationary therapy [51]. On the other hand, the device’s multimodality has allowed integrating different high-level strategies, as the methodology proposed in this paper, aiming at improving the (1) interaction, (2) motivation, (3) effort, and (4) active engagement [53].

#### 2.1.3. BCI—T-FLEX System Integration

**Figure 2:**
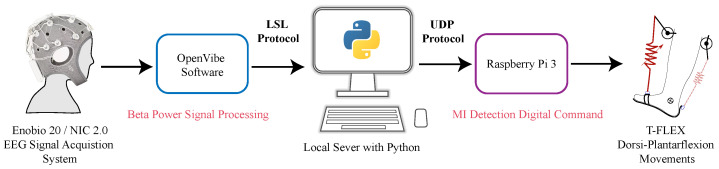
Communication protocols diagram for BCI—T-FLEX integration through a Local Server in python.

The communication bridge between the designed BCI and the T-FLEX device used different data sending protocols across a Python local server. Firstly, OpenVibe connected the local server with a Lab Streaming Layer (LSL) protocol at 256 Hz sending an array of EEG signals variables with a configured duration of 300 s (150,000 samples). The sent data included the sample number, time in seconds, channel (FCz, C1, Cz, C2, CPz), encoding type, and magnitude in terms of microvolts (uV). Subsequently, the local server processed this data to compare it with the individual threshold from the calibration stage and to remit it to the Raspberry Pi 3 employing a User Datagram Protocol (UDP). In this case, the server sent a logical ‘1’ when the threshold was exceeded and a ‘0’ when not. The above triggered or not a dorsi-plantarflexion movement assisted by the exoskeleton (see Figure 2).

#### 2.1.4. Stimuli Strategies

In addition to the previous system presented, the proposal also involved two inducement systems (i.e., visual and haptic stimulation) to generate brain incentives and facilitate the MI process.

**Figure 3:**
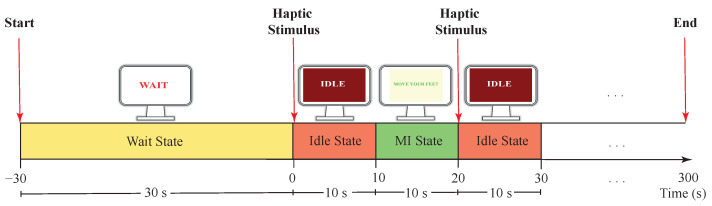
Timeline strategy applied in MI experimental conditions with visual and haptic stimulus. The Idle and MI states repeated alternately until fulfilling the 5-min test.

- Visual Stimulus System: The local server configured three types of instruction texts showed in a full-screen: (1) “Wait”, (2) “Idle”, and (3) “Move your feet”. On one hand, the main objective of the “Wait” text was to provide an initial 30 s waiting period to prepare the system. On the other hand, “Idle” and “Move your feet” texts, gave an explicit indication to the user to stay in a state of relaxation or a state of MI generation, with 10 s duration respectively (see Figure 3). In this way, only in the “Move your feet” stage, the local server received MI commands to activate T-FLEX.
- Haptic Stimulus System: The visual system worked with haptic one in sync with the “Move your Feet” periods to assist the patient in the MI generation (Figure 3). This haptic system, manually controlled by the supervisor, implemented the SunniMix rumble vibration motor (SM SunniMix, USA) with a vibration frequency in a range between 36 and 40 Hz (2200 to 2500 r/min). This motor attached the system through a structure made of Ethylene Vinyl Acetate (EVA), a box made of Acrylonitrile Butadiene Styrene (ABS) coated it, and finally, velcro material allowed the adhesion to the anterior tibialis muscle area.

### 2.2. Experimental Validation

This section exposes the experimental evaluation with the description, procedures, and methods performed to systematically compare both visual and haptic stimuli strategies.

#### 2.2.1. Participants

The development of this preliminary study considered the following inclusion and exclusion criteria for the participants’ selection:

- Inclusion Criteria: Patients between the ages range of 18 to 70 years with a pathology associated with the foot-ankle complex due to a neurological injury and with partial independence to mobilize.
- Exclusion Criteria: Candidates with hypertension, uncontrolled epilepsy, pain in the lower limbs, and severe spasticity (level 4 of the Ashworth Scale) were excluded from the study, as well as patients with the presence of wound or pressure ulcers that could have made nonfeasible the use of the device.

#### 2.2.2. Experimental Setup

**Figure 4:**
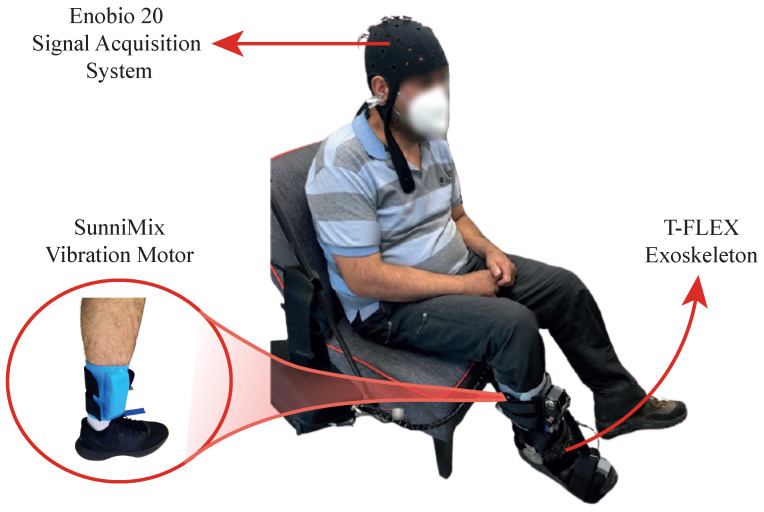
Experimental system setup for BCI-based control using T-FLEX with visual and haptic stimuli.

Participants sat comfortably in a chair with a 90° knee flexion while looking at a screen. As previously mentioned, the wireless and portable electrophysiology sensor system Enobio 20 with the NIC2 software interface established the signal acquisition system. Moreover, the T-FLEX device assisted subjects’ paretic lower limb dorsi-plantarflexion movements, and an additional motor, located in the anterior tibialis muscle area, delivered haptic stimulus during the active instants of the experimental procedure (see Figure 4).

#### 2.2.3. Experimental Procedure

**Figure 5:**
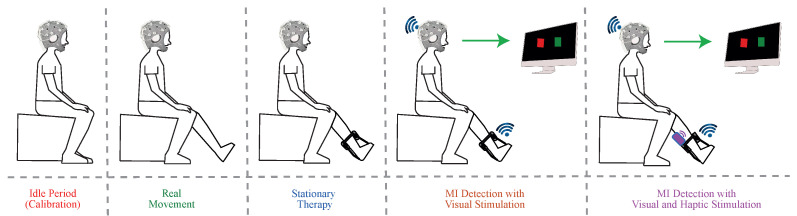
Experimental BCI T-FLEX system procedure in post-stroke patients with lowerlimb impairment.

Five captures (i.e., a calibration and four experimental conditions) guided this study with 5 min duration each one. Unlike the calibration period, the four experimental conditions considered 10 s-period alternation between active and rest intervals until reaching the 5-minute test. The first capture, Idle, referred to a calibration period that established the threshold while the user remains static. The second and third captures belonged to an experimentation period that allowed the user to become familiar with the system. In this case, while one of the tests consisted of active dorsi-plantar movements without T-FLEX assistance, the other one implied the stationary therapy (ST) assisted with the robotic device and configured to perform dorsi-plantarflexion every 3 s during the active intervals (see Figure 5).

The fourth and fifth last two captures belonged to the MI stage. Throughout the MI active intervals, subjects imagined continuous flexions and extensions movements of the ankle to command T-FLEX. In these captures, visual or visual with haptic inducement stimulated patients. That said, each participant carried out a single session to perform the test, lasting approximately 30 to 40 min.

Incidentally, physiological tonic activity in experimental settings can increase the tension of the facial muscles, generating noise in the EEG signals, especially in activities where the cognitive demand may imply a state of stress. Hence, before the beginning of the study, participants performed a training session based on Jacobson’s progressive relaxation technique for ocular, facial, jaw, and neck muscles [54]. The technique allows treating the control of reactivity to reduce anxiety and achieve a state of cognitive stability through the progressive elimination of muscular tensions [55].

In the same way, patients were instructed about the activity to perform, the eyeball fixed position to maintain on the screen during the execution of the task, and the movements to avoid (eye and body movements and contraction of facial and mandibular muscles). These strategies sought to minimize artifacts and ensure the best possible EEG signal quality.

Both training and experimental procedures were implemented by members from the Movement Analysis Laboratory of the Rehabilitation Corporation Club de Leones Cruz del Sur (Punta Arenas, Chile).

#### 2.2.4. Experimental Analysis

Both captures that employed MI (i.e., MIV and MIVH tests) evaluated the BCI system accuracy in terms of the beta rebound acquired signal. In this way, the number of active intervals in which the patient had to imagine movement was related to the MI attempts correctly detected by the BCI, following Equation (2). As soon as the 10 s active period started, with the visual interface “Move your feet”, the system detected a successful attempt once the beta rebound power exceeded the threshold. In this way, it was expected to measure the first successful attempt per each active window (total of 14 active periods). Statistical analyses verified significant differences between visual stimulation and visual and haptic stimulation.

(2) $$\mathrm{Accuracy}(\%)=\frac{\mathrm{Successfull}\;\mathrm{Attempts}}{\mathrm{Total}\;\mathrm{Attempts}}\times 100\;$$

In terms of the brain-motor activity, the power spectral density (PSD) estimated the variation of energy inside the Event-Related Potential (ERP), which measured the electrical response after integrating the BCI system with both inducement strategies. Firstly, each continuous offline signal passed through a high-pass filter of 0.16 Hz to remove slow drifts and through a 4th-order Butterworth band-pass filter to segment the MI frequency band (8–30 Hz). Then, the signal was segmented into the 10-second active periods and squared to obtain power samples. The total segments of each task were averaged and adjusted through a baseline correction by subtracting the mean activity 500 ms before the stimuli. Equation (3) demonstrates the procedure, where P(t) is the average power signal of the active periods and $P_{Baseline}$ is the average power number during the last 500 ms of the rest periods [19,20,56,57].

(3) $$ERP_{channel}=P\left(t\right)-P_{Baseline}\;$$

Afterward, the Welch method used a window of 0.5 s and 50% overlapping to obtain the PSD in the frequency band associated with the MI of each channel. Statistical analysis checked whether there were significant differences in the PSDs associated with each patients’ channel ERP for the ST, MIV and MIVH states.

At least the Quebec User Evaluation of Satisfaction with Assistive Technology (QUEST) survey tests determined patients’ satisfaction level with the device. This information functioned as feedback from the user, regarding the operation and structure of the proposed system.

## 3. Results

### 3.1. Participants

The study included five patients (56.24 ± 3.26 years old) who presented a lower limb hemiparesis due to a cerebrovascular accident (see Table 1). All subjects successfully performed the tasks without reporting fatigue, stress or anxiety during the experiment.

### 3.2. Accuracy Results

**Figure 6:**
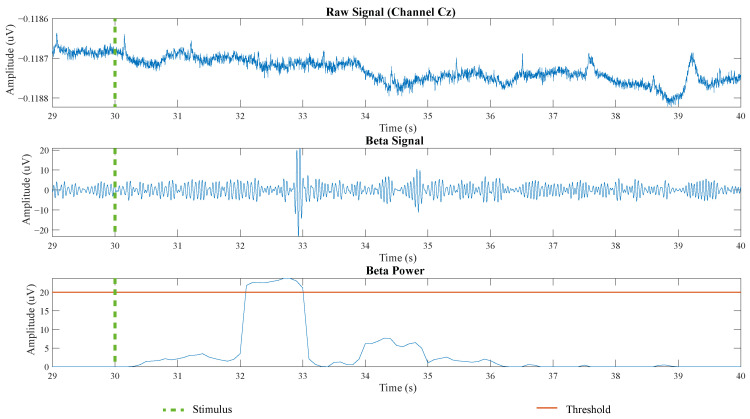
Processing results over 10 s of an MIVH active period to detect the beta rebound signal. First, the raw signal over channel Cz appears. The second signal refers to the filtered wave in the beta frequency band (16–24 Hz). The last signal shows the beta signal squared and averaged compared with the threshold (horizontal orange line). The dotted and vertical green line refers to the moment in which the stimulus was given.

As previously mentioned, the accuracy results were based on quantifying the number of times the beta wave rebound power exceeded the threshold throughout the session. Figure 6 displays the signal processing result to take the beta rebound power as a command to trigger the T-FLEX dorsiflexion and plantarflexion movements.

In this case, two events occurred where the signal power increased for approximately 1 s. However, the system only detected the command when the amplitude exceeded the threshold, which happened 2 s after the command “Move your feet” was visually and haptically given to the patient. It is important to emphasize only the first beta power that exceeded the threshold was relevant to control the robotic device.

Now, from the beta power and the threshold, it was possible to establish the successful attempts in the motor imagination of flexion and extension movements of the ankle. Table 2 displays the result of the threshold value and average detection time after the active MI periods stimuli. There, subject 4 had the highest threshold level and also one of the best results in the time detection average for both paradigms. In general, MI detection based on the beta rebound occurred between 986 ms and 3326 ms after the stimuli strategies. Furthermore, most patients performed better in time during the MIVH than for the MIV test.

**Figure 7:**
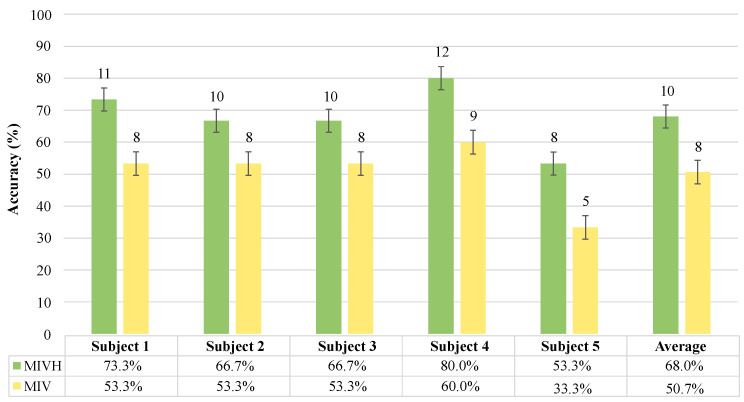
Accuracy results of Motor Imagery (MI) detection for each patient in the Motor Imagery with Visual and Haptic (MIVH) stimuli test in green and the Motor Imagery with Visual (MIV) stimulus test in yellow. Each bar graph presents in its upper side the number of MI attempts achieved over the 15 opportunities presented throughout each of the two stages. The last two bars to the right side are the average of the five subjects’ accuracy.

In terms of accurate results for both MI detection with visual stimulation (MIV) and visual and haptic stimulation (MIVH), Figure 7 shows the compiled results in a bar graph grouped by the patient. In green the accuracy calculated for the MIVH test and, in yellow, the accuracy calculated for the MIV test can be seen. According to all subjects’ results, the BCI performance on motor imagery was better based on visual and haptic stimulation. The performance average accuracy in the case of the visual stimulus was ranging 50.7% with a mean of 8 attempts, while the visual and haptic strategy was around 68% with 10 over 15 opportunities. Moreover, as shown in Figure 7, the highest accuracy was present in both paradigms for subject 4 while the worst was performed by subject 5. Overall, although MI detection was efficient for active periods, abrupt increases in the beta power were also found during a total average of 5 rest events for both paradigms where the robotic device was previously conditioned for a non-activation, according to the stimulation protocol.

Shapiro–Wilk normality results test showed a normal distribution of the data for the MIV test and a non-normal distribution for MIVH (*p* > 0.05). Therefore, the non-parametric Wilcoxon test used to compare two related samples indicated a 0.038 *p*-value over a 0.05 significance level (*p* < 0.05). In this case, the null hypothesis was rejected. Accordingly, there were significant differences in the accuracies medians of the patients’ MIV and MIVH tests.

### 3.3. Power Spectral Density Results

In terms of the EEG offline analysis, Table 3 presents the PSD mean and standard deviation for the MI frequency band (8–30 Hz). The Cpz channel of patient 4 was discarded due to its low data quality that prevented the extraction of functional characteristics.

**Figure 8:**
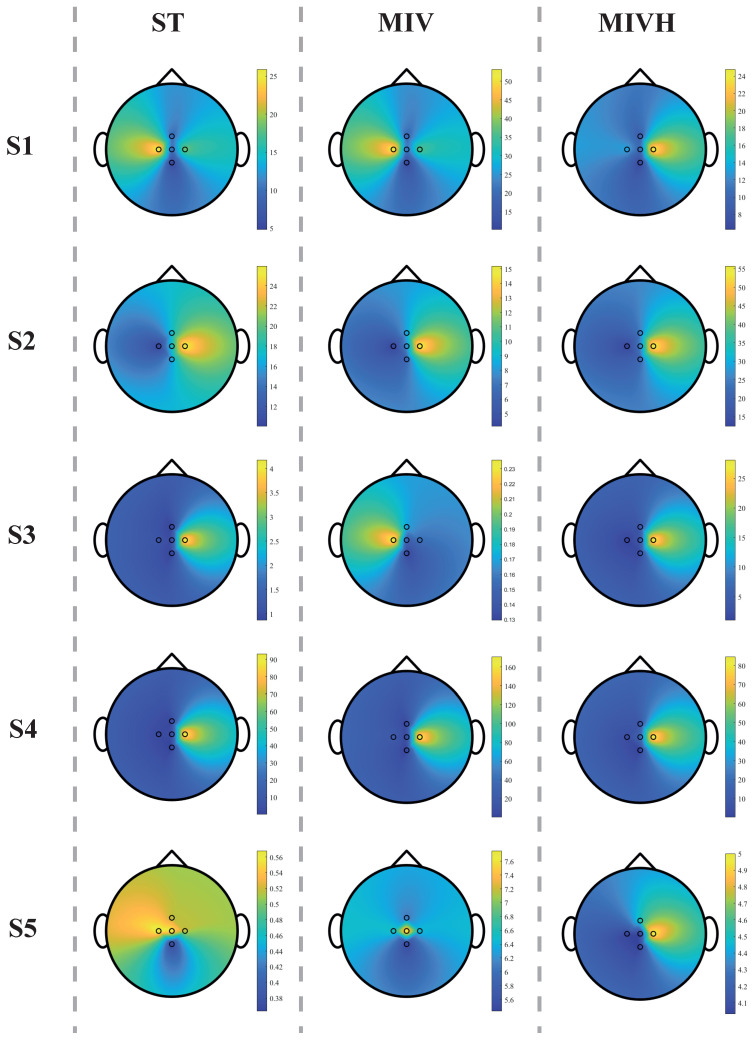
Electroencephalography (EEG) topographies of Power Spectral Density (PSD) associated with the Event-Related Potentials (ERPs) of all patients tests.

Topographies better illustrated PSD characteristics associated with the event-related potential of each user channel (see Figure 8). Visually, topographic maps revealed in most patients similar brain activity for the three paradigms with a remarkable activity over the left and right sides. Subjects 1, 3, and 4 had a right paretic side, of which all of them presented not persistent laterality changes in all the paradigms. Only subject 2 demonstrated cerebral activity associated with the contralateral MI in all stages. In this way, this patient easily performed movement imagination over his left paretic limb [20,48]. In contrast, subject 4 evidenced an ipsilateral performance to his right paretic side. These results were not expected according to the patients’ motor imagery accuracy execution (Figure 7), where subject 4 presented significantly better performance results.

The Shapiro–Wilk test analyzed the normality for each channel used in this study. Specifically, channels C2 and Cpz did not present a normal distribution (*p* > 0.05) for MIV and MIVH tests, respectively. The other trials followed a normal distribution. Subsequently, the statistical test determined significant changes between the assessed modalities, i.e., ST, MIV, and MIVH. In this way, a Wilcox and t-student test analyzed the three groups in terms of the user’s channel PSD mean, considering the data normality. These trials exhibited no statistical changes between the assessed groups (*p* < 0.05). Therefore, the groups were compared through the ANOVA and Friedman tests as follows: (1) the ST and MIV tests, (2) the ST and MIVH tests, and (3) the MIV and MIVH tests. As seen in Table 4, only the channel Fcz had significant differences for the ST compared to MIVH tests (*p* < 0.05).

### 3.4. User Perception Results

Lastly, as mentioned in the methodological section, a survey evaluated the user perception of the designed BCI system. Table 5 summarizes the results obtained for each of the users. The final results were averaged to know the general results, where the satisfaction level was between satisfied and strongly satisfied. The total average QUEST score of all patients was 4.76 and for the extending QUEST version (i.e., including reliability, speed, learning, and aesthetic design) the score was 4.55. From the users’ perspective, adjustment, ease of use, and reliability criteria were the relevant aspects selected through the QUEST survey.

## 4. Discussion

The results of the proposed system demonstrated the viability of acquiring the beta rebound signal to command the exoskeleton. Subjects successfully performed all tasks where mental ability was required with a good performance accuracy in visual or visual with haptic stimulation. However, neither generated differential electrical activity throughout the session stages.

As an initial approach, the algorithm for real-time detection of MI was ideal as a proof of concept in the BCI-based device control. The calibration strategy made it possible to individually adapt and define the user’s basal level of brain activity at which changes in beta rebound were detected. Moreover, the thresholding technique further limited MI detection for random events resulting from even haptic stimulation. Nevertheless, analysis of the offline signals demonstrated imagery activity during passive or non-imagery events. This behavior is related as a consequence of the user’s lack of concentration or as a leftover effect from the active state. In any case, the results suggest the need to establish longer intermediate times to pass from one state to another, and a much more robust machine-learning algorithm to guarantee the beta rebound detection in more complex contexts.

On the other hand, the most relevant MI accuracy results of this study showed an average difference of 17.3% of the MIVH test over MIV active periods. All patients had a greater number of successful attempts at motor imagery when haptic stimulation was present. These results were related to the proprioceptive delivery, to the CNS, of a neural representation or mimic of the natural body mechanics to facilitate the creation of the movement mental image [39,58,59]. In contrast, both topographically and statistically in PSD results revealed no significant differences between the stationary therapy without MI, and the MI with the visual and visual-haptic stimulus strategies. The above data suggest visual and haptic stimuli improve the subject’s accuracy in performing motor imagery but do not generate differential brain activity. Similar results were reported by Kauhanen et al. [43], who did not report differences between haptic or visual stimuli with upper limb exoskeleton. In the case of the present study, only the Cpz channel in the ST and MIVH tests presented significant differences, indicating the neural variations during cognitive tasks and the activation of the somatic sensory association area as a result of the haptic stimulation [60,61].

Furthermore, within these results, it was also possible to identify data variability in terms of detection time, accuracy, and PSD power. As could be observed, the comparison performance between subjects 4 and 5 was the opposite, and in general, each participant revelated variable results. Initially, it could be associated with environmental or clinical variables that vary between patients (e.g., age, stroke year, and even the type of treatment). However, it is mostly related to intersubject variability in the motor imagery performing, where not all participants have the same facility to create a visual image of a movement. Emami et al. [31], have made findings of the significant role of the distractor factors affecting the MI-BCI performance results.

In the same way, topographical maps showed potential changes over the contralateral and the ipsilateral hemisphere. Although the proposed BCI-controlled exoskeleton system did not contemplate the beta rebound laterality for the device control, the offline analysis over alfa and beta signals (8–32 Hz) exhibited a significant impact on the C1 and C2 channels during the active periods of MI (Figure 8). In this case, the movement imagery-related lateralization had a higher discriminative power over the imagery of left foot movement (i.e., C2 channel). However, only two of the five patients had left hemisphere involvement. This way, the remaining participants presented, in some cases, a compensatory effect of the healthy side on the loss of functions of the paretic side. This behavior could have the opposite consequences to those expected with the BCI system, whose emphasis should be on the recovery of the paretic hemisphere at the neuronal and motor level. Therefore, training approaches should be considered involving the cerebral-affected side in BCI strategies.

Now, according to the results presented in Table 5, a favorable result was concluded regarding user perception. Besides, no patient exhibited affectations in the locomotor system, pressure points, fatigue, stress, or anxiety during the experimental procedure. Within the assessment, the rehabilitation technology weight and the instructions at the time of use were remarkable for patients. Moreover, the reliability and the easiness of the learning process were optimal according to the patients’ perception in the extended test due to the simple versions of the task. This last criterion was beneficial since one of the most encountered problems in current BCI systems with neurological patients was the task learning system [8]. According to Zickler et al. [62], this result was helpful for the study in general, since this designed technology is aimed at rehabilitation. Therefore, it is conclusive that the technology complied with a sufficient design for the use of patients. This survey did not eliminate some of the shortcomings previously presented, but it did contribute to subjective patient satisfaction, which may benefit possible long-term studies with T-FLEX.

One of the strengths of this preliminary study is the system integrated strategy to command the portable and low-cost T-FLEX exoskeleton with inter-device connection strategies, relaxation tactics before the experimental session, and straightforward stimulus strategies. However, it was limited in terms of the number of patients, the number of sessions, and the number of mapped channels for offline EEG analysis, from which it was not possible to find statistically significant differences between the stimuli approach.

## 5. Conclusions

This study presents the BCI integration system to the T-FLEX lower-limb exoskeleton combining two different stimulus modes for post-stroke patients. The experimental results demonstrated the proposed system’s ability to detect MI with an increase on average from 50.7% to 68% when the stimulus was not only visual. Nevertheless, no significant differences were found in the PSD mean of active periods between the ST, MIV, and MIVH tests. Only the Cpz channel appeared to represent differences in ST and MIVH tests related to the sensorial cue and the higher neural activity required during the MI process. In addition, PSD topographic maps showed the contralateral MI activity, which was indispensable to demonstrate the intrasubject variability and the healthy hemisphere response.

In terms of the user’s subjective perception, the BCI system implementation is viable since has a good acceptance. However, deeper and long-term assessments monitoring correlations between muscle and brain activity are required to allow evidence about neuroplasticity induction. Future works should focus on additional data processing and classification procedures to better quantify beta rebound power activity in more complex contexts and considering the MI laterality over the affected side. Likewise, the assessment of the BCI-controlled ankle exoskeleton system in long-term sessions with a more extensive sample of post-stroke patients is indispensable to evaluate the efficiency and effect of the system in a broad spectrum. Moreover, in a larger scope, stimuli with informational and additional feedback strategies should be implemented to improve the MI performance sought by BCI systems.

## Figures and Tables

**Table 1:** Subjects’ clinical information.

Subject	Age (Years)	Weight (Kg)	Height (cm)	BMI	Paretic Side
1	55	84	173	28.1	Right
2	62	96	168	34	Left
3	63	79	161	30.5	Right
4	56	94	164	34.9	Right
5	61	69	166	25	Left

**Table 2:** Subjects’ threshold results with the average detection time in MIV and MIVH strategies.

Subject	Threshold	MIV Detection Time	MIVH Detection Time
1	8 uV	1798 ms	2116 ms
2	8 uV	1272 ms	1173 ms
3	4 uV	3326 ms	1646 ms
4	20 uV	1226 ms	1010 ms
5	12 uV	2418 ms	986 ms

**Table 3:** Descriptive PSD statistics associated with each patient channel for the ST, MIV, and MIVH test. * Not Applied: The Cpz channel of subject 4 did not have the necessary signal quality to be included in the studies.

PSD (dB/Hz) Mean						
Test	Subject	Fcz	C1	Cz	C2	Cpz
ST	1	9.14	26.00	9.41	18.70	4.74
	2	17.38	9.99	17.85	26.03	15.98
	3	0.86	1.12	0.97	4.20	0.86
	4	1.51	2.06	2.76	93.78	NA *
	5	0.52	0.57	0.56	0.52	0.36
MIV	1	18.30	53.39	20.89	36.79	10.03
	2	8.26	4.10	7.67	15.25	6.36
	3	0.17	0.24	0.15	0.16	0.13
	4	2.19	20.03	4.25	172.11	NA *
	5	6.07	6.55	7.78	6.47	5.42
MIVH	1	8.98	13.30	10.17	24.89	6.19
	2	24.45	12.35	24.89	55.95	21.41
	3	3.43	0.96	3.52	28.29	1.69
	4	7.95	2.99	16.55	85.16	NA *
	5	4.52	4.04	4.04	5.00	4.03
ST	1	9.67	27.83	10.57	19.63	5.07
	2	19.65	11.95	20.28	31.00	18.51
	3	1.05	1.15	1.11	4.29	1.05
	4	1.73	2.49	3.11	121.08	NA *
	5	0.79	0.80	0.90	0.81	0.51
MIV	1	19.56	55.74	23.22	38.74	11.23
	2	9.85	4.86	9.04	19.86	7.34
	3	0.18	0.29	0.17	0.19	0.15
	4	2.56	25.21	5.24	185.08	NA *
	5	8.86	10.06	11.75	9.84	8.02
MIVH	1	9.91	13.26	10.96	26.81	7.31
	2	21.93	13.43	21.76	65.34	24.11
	3	4.13	1.24	4.77	33.19	2.24
	4	7.62	2.92	18.40	110.69	NA *
	5	4.30	4.38	3.97	4.78	3.63

**Table 4:** *p*-Values along the comparison of ST, MIV and MIVH paradigms. The green values indicate significant statistical results.

Test Comparison	Fcz	C1	Cz	C2	Cpz
ST vs. MIV vs. MIVH	0.704	0.498	0.562	0.549	0.368
ST vs. MIV	0.737	0.218	0.645	0.437	0.999
ST vs. MIVH	0.039	0.699	0.074	0.184	0.100
MI vs. MIVH	0.532	0.300	0.509	0.999	0.530

**Table 5:** Collecting QUEST survey results with extending additional criteria. The highlighted values with green refer to the better outcomes.

QUEST Survey Responses						
Criteria	S1	S2	S3	S4	S5	Average
Dimensions	4.00	5.00	3.00	4.00	5.00	4.20
Weight	5.00	5.00	5.00	5.00	5.00	5.00
Adjustment	5.00	5.00	4.00	5.00	5.00	4.75
Safety	5.00	5.00	4.00	5.00	5.00	4.75
Ease of use	5.00	5.00	5.00	4.00	5.00	4.75
Effectiveness	5.00	5.00	5.00	4.00	5.00	4.75
Information/Instuctions	5.00	5.00	5.00	5.00	5.00	5.00
**QUEST Total Score**	**4.85**	**5.00**	**4.42**	**4.57**	**5.00**	**4.76**
**Extended QUEST Survey Responses**						
Reliability	5.00	5.00	4.00	5.00	5.00	4.75
Speed	4.00	4.00	4.00	4.00	4.00	4.00
Learning	4.00	5.00	5.00	5.00	5.00	4.75
Aesthetic design	4.00	5.00	4.00	5.00	5.00	4.25
**Added Items Total Score**	**4.25**	**4.75**	**4.25**	**4.75**	**4.75**	**4.55**

## Data Availability

This study was registered as BCI-Based Control for Ankle Exoskeleton T-FLEX: Comparison of Visual and Haptic Feedback With Stroke Survivors on 6 August 2021 in Clinical Trials with the identifier No. NCT04995367 (available at https://www.clinicaltrials.gov/ct2/show/NCT04995367) (accessed on 6 August 2021).

## Abbreviations

The following abbreviations are used in this manuscript:

BCI	Brain-Computer Interface
EEG	Electroencephalography
MI	Motor Imagination
MIV	Motor Imagination with Visual Stimulus
MIVH	Motor Imagination with Visual and Haptic Stimuli
ERP	Event-Related Potential
PSD	Power Spectral Density
ERS/ERD	Event-Related Desynchronization/Synchronization
